# S100A gene family: immune-related prognostic biomarkers and therapeutic targets for low-grade glioma

**DOI:** 10.18632/aging.203103

**Published:** 2021-06-08

**Authors:** Yu Zhang, Xin Yang, Xiao-Lin Zhu, Hao Bai, Zhuang-Zhuang Wang, Jun-Jie Zhang, Chun-Yan Hao, Hu-Bin Duan

**Affiliations:** 1 Department of Neurosurgery, First Hospital of Shanxi Medical University, Taiyuan 030001, Shanxi, P.R. China; 2 Department of Geriatrics, First Hospital of Shanxi Medical University, Taiyuan 030001, Shanxi, P.R. China; 3 Department of Neurosurgery, Lvliang People's Hospital, Lvliang 033000, Shanxi, P.R. China

**Keywords:** S100A, low-grade glioma, prognosis, immune, mutation

## Abstract

Background: Despite the better prognosis given by surgical resection and chemotherapy in low-grade glioma (LGG), progressive transformation is still a huge concern. In this case, the S100A gene family, being capable of regulating inflammatory responses, can promote tumor development.

Methods: The analysis was carried out via ONCOMINE, GEPIA, cBioPortal, String, GeneMANIA, WebGestalt, LinkedOmics, TIMER, CGGA, R 4.0.2 and immunohistochemistry.

Results: S100A2, S100A6, S100A10, S100A11, and S100A16 were up-regulated and S100A1 and S100A13 were down-regulated in LGG compared to normal tissues. S100A3, S100A4, S100A8, and S100A9 expression was up-regulated during the progression of glioma grade. In addition, genetic variation of the S100A family was high in LGG, and the S100A family genes mostly function through IL-17 signaling pathway, S100 binding protein, and inflammatory responses. The TIMER database also revealed a relationship between gene expression and immune cell infiltration. High expression of S100A2, S100A3, S100A4, S100A6, S100A8, S100A9, S100A10, S100A11, S100A13, and S100A16 was significantly associated with poor prognosis in LGG patients. S100A family genes S100A2, S100A3, S100A6, S100A10, and S100A11 may be prognosis-related genes in LGG, and were significantly associated with IDH mutation and 1p19q codeletion. The immunohistochemical staining results also confirmed that S100A2, S100A3, S100A6, S100A10, and S100A11 expression was upregulated in LGG.

Conclusion: The S100A family plays a vital role in LGG pathogenesis, presumably facilitating LGG progression via modulating inflammatory state and immune cell infiltration.

## INTRODUCTION

Glioma is the most common type of intracranial tumor. Low-grade glioma (LGG) is classified by the World Health Organization (WHO) into grade I and grade II [1]. Currently, LGG is primarily treated with surgical resection, radiotherapy, and chemotherapy, having a relatively favorable prognosis [2–4]. However, in the process of glioma development, mutated genes accumulate and the tumor microenvironment changes, and LGGs may slowly develop into high-grade gliomas (HGGs) [5–8]. Macrophages release inflammatory substances that cause gliomas to appear more aggressive [9, 10]. In line with this evidence, there is a hypothesis that regulation of the tumor immune process may potentially play a role in the treatment of glioma [11]. However, there are currently many challenges in glioma immunotherapy. For example, gliomas themselves secrete inhibitory cytokines, which leads to an immunosuppressive microenvironment [12, 13]. Moreover, the immune resistance of tumor cells in gliomas is significantly increased, and immune cells may transition from an anti-tumor phenotype to pro-tumor phenotype [14, 15]. Therefore, it is necessary to develop appropriate targets as the operational points of glioma treatment. Secretion of S100A protein is detectable in the extracellular space and in certain body fluids, such as serum, urine, sputum, cerebrospinal fluid, and feces [16–18]. S100A family members are widely involved in a variety of inflammatory disease regulatory processes, including ischemic heart inflammation, Kawasaki disease, eye inflammation, and chorioamnionitis, among others [19–23]. Multiple studies have shown that some of the S100A family members regulate tumor development by mediating tumor immune processes [24–27]. Therefore, we investigated the role of the S100A protein family in glioma pathology.

## RESULTS

### Differential expression of S100A family genes in LGG

**Figure 1:**
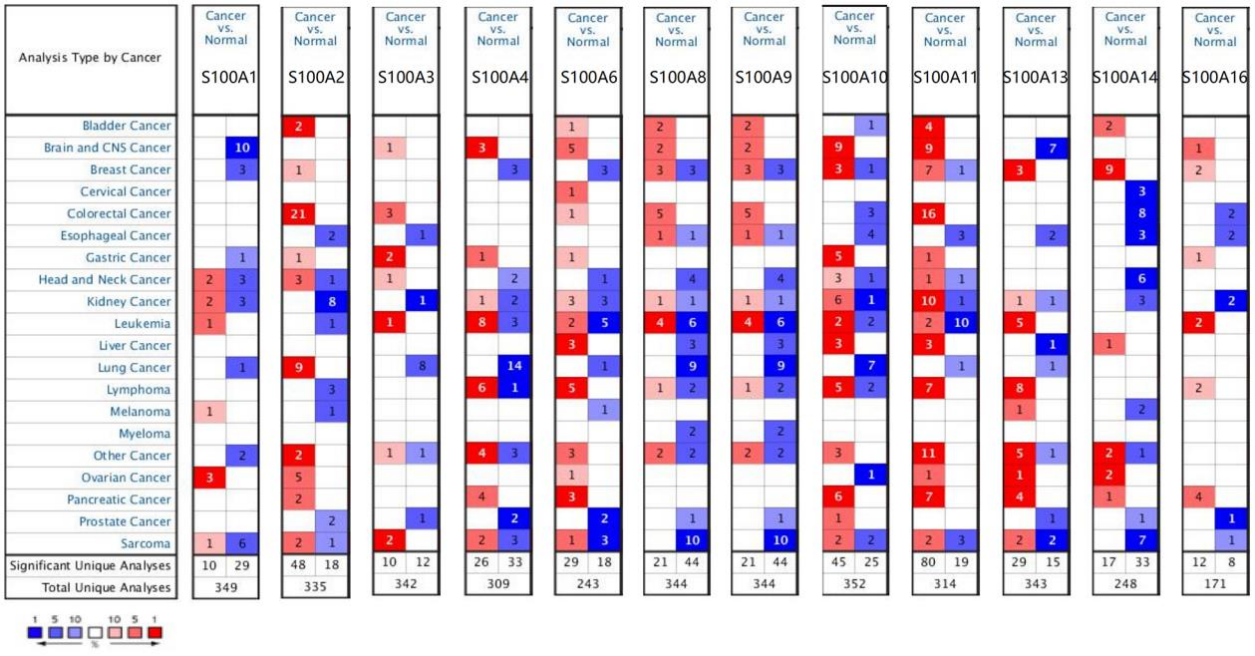
**Differential expression of S100A family genes in various types of tumors and normal tissues.** Red: high expression level, darker color means higher expression level, blue: low expression level, darker color means lower expression level.

We retrieved 12 S100A family genes, including S100A1, S100A2, S100A3, S100A4, S100A6, S100A8, S100A9, S100A10, S100A11, S100A13, S100A14, and S100A16, and acquired their mRNA expression levels in various tumors and normal tissues via the ONCOMINE database. Results showed that compared to normal tissues, the expression of S100A3, S100A4, S100A6, S100A8, S100A9, S100A10, S100A11, and S100A16 was upregulated in CNS cancer tissues, while S100A1 and S100A13 were downregulated (Figure 1).

**Figure 2:**
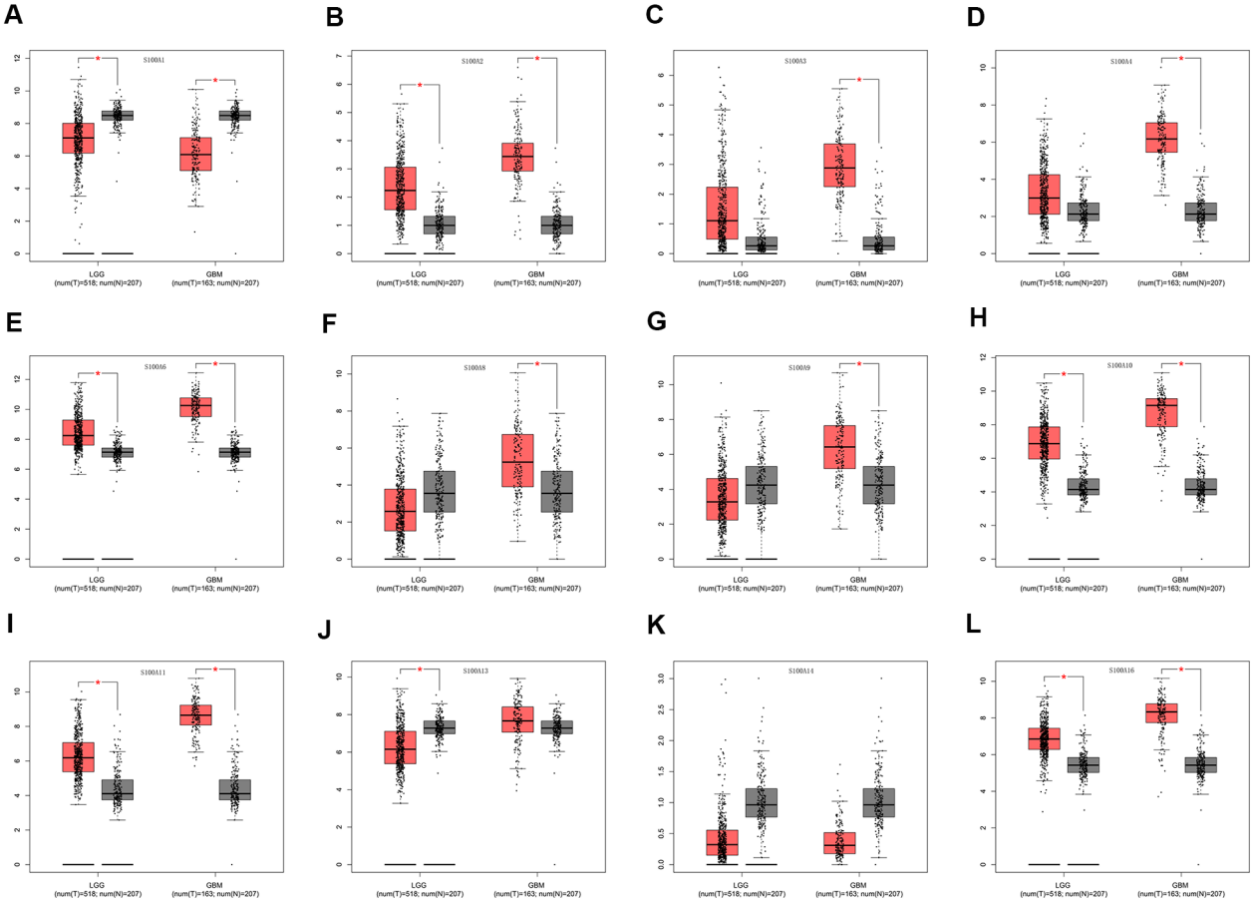
**Differential expression of S100A family genes in LGG, GBM and normal tissues.** (**A**) S100A1, (**B**) S100A2, (**C**) S100A3, (**D**) S100A4, (**E**) S100A6, (**F**) S100A8, (**G**) S100A9, (**H**) S100A10, (**I**) S100A11, (**J**) S100A13, (**K**) S100A14, (**L**) S100A16. *P < 0.05.

We assessed differential expression of 12 S100A family genes across LGG, GBM, and normal tissues via the GEPIA database Single Gene Analysis module. The results showed that S100A2, S100A6, S100A10, S100A11, and S100A16 were upregulated and S100A1 and S100A13 were downregulated in LGG compared to normal tissues (p<0.05). S100A2, S100A3, S100A4, S100A6, S100A8, S100A9, S100A10, S100A11, and S100A16 were upregulated and S100A1 was downregulated in GBM compared to normal tissues (p<0.05). There was no significant difference in the expression of S100A3, S100A4, S100A8, and S100A9 in LGGs compared with normal tissues. However, expression of these genes was upregulated during the progression of glioma grade (Figure 2A–2L).

### Gene mutations and PPI networks of S100A family in LGG

**Figure 3:**
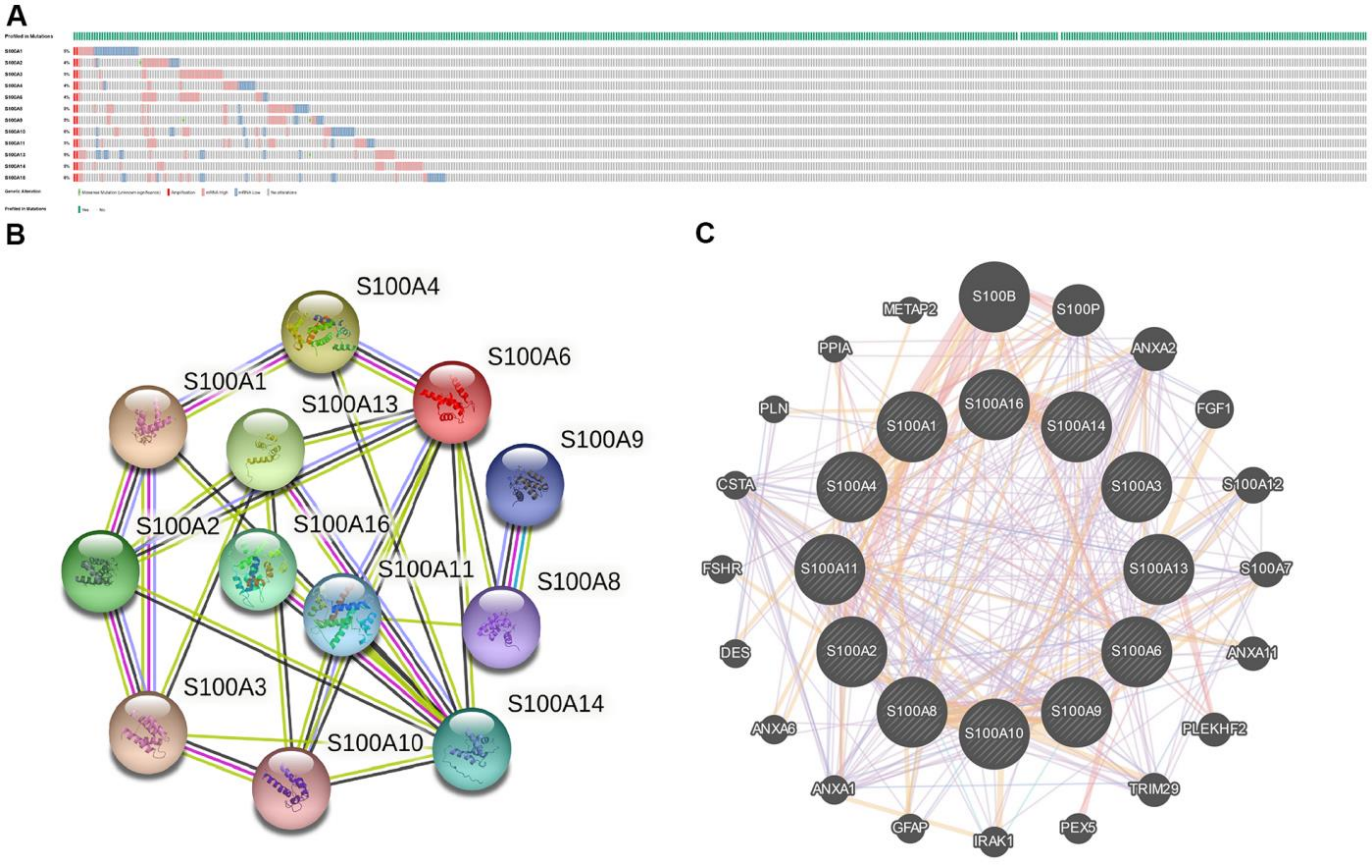
(**A**) Gene mutations of S100A family in LGG; (**B**) PPI networks for S100A family genes in the String database; (**C**) PPI network of S100A family genes in the GeneMANIA database.

Via the cBioPortal database, we analyzed genetic variation of the S100A family based on the LGG sample data from the TCGA database. The results are presented in Figure 3A. The mutation rate was 4% for S100A2, S100A4, and S100A6, 5% for S100A1, S100A3, S100A8, S100A9, S100A11, S100A13, and S100A14, and 6% for S100A10 and S100A16.

We also performed PPI network analysis of the S100A family via STRING and GeneMANIA to explore potential protein interactions and to seek network-related genes. The PPI network identified by the STRING database presented 12 S100A family gene nodes and 26 edges (Figure 3B). S100A family gene functions were suggested to be related to the IL-17 signaling pathway, S100 protein binding, and inflammatory responses. Furthermore, the GeneMANIA results also showed that the function of S100A family genes (S100A1, S100A2, S100A3, S100A4, S100A6, S100A8, S100A9, S100A10, S100A11, S100A13, S100A14, S100A16) were mainly associated with S100 protein binding, calcium ion binding, and calcium-dependent protein binding. S100A family protein network-related genes included S100B, S100P, ANXA2, FGF1, S100A12. S100A7, ANXA11, PLEKHF2, TRIM29, PEX5, IRAK1, GFAP, ANXA1, ANXA6, DES, FSHR, CSTA, PLN, PPIA, and METAP2 (Figure 3C).

### Enrichment analysis of S100A family genes in LGGs

**Figure 4:**
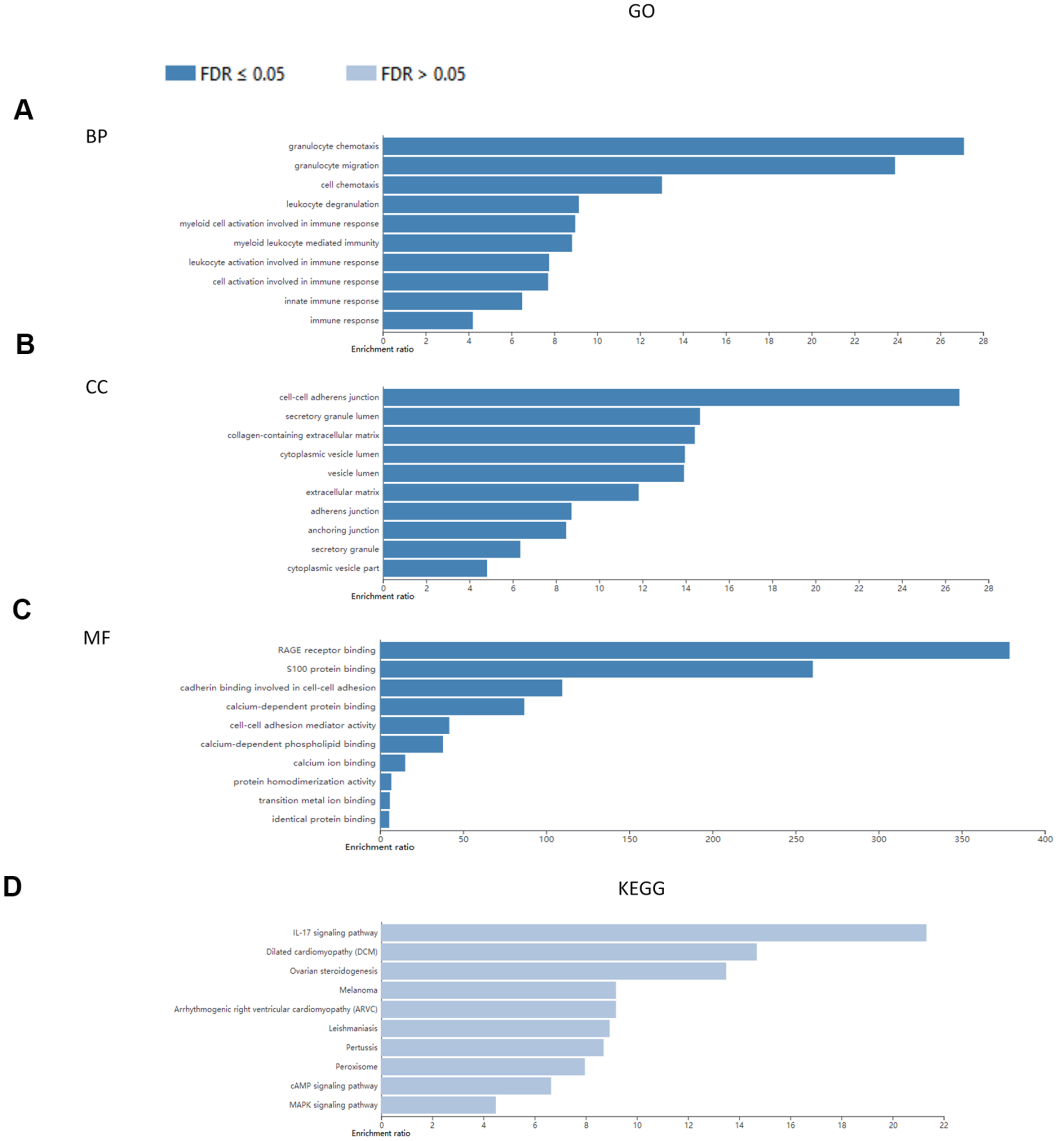
**Enrichment analysis of S100A family genes and protein network-associated genes.** (**A**) BP; (**B**) CC; (**C**) MF; (**D**) KEGG.

We conducted GO functional annotation and KEGG pathway enrichment analysis of S100A family genes (S100A1, S100A2, S100A3, S100A4, S100A6, S100A8, S100A9, S100A10, S100A11, S100A13, S100A14, S100A16) and protein network-related genes (S100B, S100P, ANXA2, FGF1, S100A12, S100A7, ANXA11, PLEKHF2, TRIM29, PEX5, IRAK1, GFAP, ANXA1, ANXA6, DES, FSHR, CSTA, PLN, PPIA, METAP2) based on the WebGestalt database (Table 1). The results show that at the BP level, these genes were mainly enriched in granulocyte chemotaxis, granulocyte migration, leukocyte activation involved in immune response, cell activation involved in immune response, leukocyte degranulation, myeloid cell activation involved in immune response, immune response, myeloid leukocyte mediated immunity, cell chemotaxis, and innate immune response (Figure 4A). At the CC level, the genes were predominantly enriched in extracellular matrix, collagen-containing extracellular matrix, secretory granule lumen, cytoplasmic vesicle lumen, vesicle lumen, cell-cell adherens junction, cytoplasmic vesicle part, adherens junction, anchoring junction, and secretory granule (Figure 4B). At the MF level, the genes were enriched in calcium ion binding, calcium-dependent protein binding, RAGE receptor binding, S100 protein binding, identical protein binding, cadherin binding involved in cell-cell adhesion, protein homodimerization activity, transition metal ion binding, cell-cell adhesion mediator activity, and calcium-dependent phospholipid binding (Figure 4C). No significant enrichment was detected in the KEGG pathway (all FDR>0.05), though IL-17 was the main highly enriched signaling pathway (Figure 4D).

**Table 1:** Enrichment analysis of S100A family genes and protein network-associated genes.

**Category**	**Term**	**Description**	**Ratio**	**P-value**	**FDR**
BP term	GO:0071621	granulocyte chemotaxis	27.103	7.43E-08	0.00050541
BP term	GO:0097530	granulocyte migration	23.891	1.58E-07	0.00050541
BP term	GO:0002366	leukocyte activation involved in immune response	7.7568	2.91E-07	0.00050541
BP term	GO:0002263	cell activation involved in immune response	7.7123	3.07E-07	0.00050541
BP term	GO:0043299	leukocyte degranulation	9.1454	3.31E-07	0.00050541
BP term	GO:0002275	myeloid cell activation involved in immune response	8.9758	3.87E-07	0.00050541
BP term	GO:0006955	immune response	4.2018	3.90E-07	0.00050541
BP term	GO:0002444	myeloid leukocyte mediated immunity	8.8283	4.45E-07	0.00050541
BP term	GO:0060326	cell chemotaxis	13.02	8.08E-07	0.00081587
BP term	GO:0045087	innate immune response	6.5	1.4691E-06	0.0011369
CC term	GO:0031012	extracellular matrix	11.823	5.25E-09	0.000003782
CC term	GO:0062023	collagen-containing extracellular matrix	14.42	6.44E-09	0.000003782
CC term	GO:0034774	secretory granule lumen	14.661	4.54E-08	0.000015891
CC term	GO:0060205	cytoplasmic vesicle lumen	13.963	6.61E-08	0.000015891
CC term	GO:0031983	vesicle lumen	13.921	6.76E-08	0.000015891
CC term	GO:0005913	cell-cell adherens junction	26.656	0.000001097	0.00021484
CC term	GO:0044433	cytoplasmic vesicle part	4.8134	2.1766E-06	0.00034453
CC term	GO:0005912	adherens junction	8.7202	2.3457E-06	0.00034453
CC term	GO:0070161	anchoring junction	8.4684	2.9165E-06	0.00038077
CC term	GO:0030141	secretory granule	6.3513	6.5396E-06	0.0007684
MF term	GO:0005509	calcium ion binding	15.164	0	0
MF term	GO:0048306	calcium-dependent protein binding	86.813	0	0
MF term	GO:0050786	RAGE receptor binding	378.82	0	0
MF term	GO:0044548	S100 protein binding	260.44	2.22E-16	1.04E-13
MF term	GO:0042802	identical protein binding	5.5282	1.46E-10	5.49E-08
MF term	GO:0098641	cadherin binding involved in cell-cell adhesion	109.66	4.25E-08	0.000013292
MF term	GO:0042803	protein homodimerization activity	6.8129	2.46E-07	0.000064984
MF term	GO:0046914	transition metal ion binding	5.9078	2.77E-07	0.000064984
MF term	GO:0098632	cell-cell adhesion mediator activity	41.67	2.4215E-06	0.00050501
MF term	GO:0005544	calcium-dependent phospholipid binding	37.882	0.000003562	0.00066858
KEGG_PATHWAY	hsa04657	IL-17 signaling pathway	21.323	0.00031034	0.10117

### Kinase target, transcription factor target, miRNA target of S100A family genes in LGG

From GSEA analysis of the LinkedOmics database, we observed that PRKDC was the top kinase target for S100A1, LYN was the main kinase target for S100A2, S100A3, S100A8, S100A10, and S100A11, LCK was the kinase target for S100A4, S100A6 and S100A9, and CDK2, ATR, and ATM were the kinases targets for S100A13, S100A14, and S100A16. The transcription factor target of S100A1, S100A2, S100A6, S100A8 and S100A9 was NRSF. The main transcription factor target of S100A3 and S100A4 was SRF. The main transcription factor target of S100A10 and S100A11 was IRF. And S100A13, S100A14 and S100A16 were respectively corresponding to MAX, E2F and YY1. The top miRNA targets for S100A1, S100A2, S100A3, S100A8, S100A9, S100A10, S100A13, S100A14 were miR-490, miR-369-3P, miR-500, (miR-517A, miR-517C), miR-409-5P, miR-33, (miR-193A, miR-193B), and miR-129. The primary miRNA target for S100A4 and S100A11 was miR-34B, and miR-9 for S100A6 and 100A16 (Table 2).

**Table 2:** Kinase target, transcription factor target, miRNA target of S100A family genes in LGG.

**Gene symbol**	**Enriched category**	**Geneset**	**LeadingEdgeNum**	**FDR**
S100A1	Kinase Target	Kinase_PRKDC	22	0
	Transcription Factor Target	V$NRSF_01	39	0
	miRNA Target	CCAGGTT,MIR-490	27	0
S100A2	Kinase Target	Kinase_LYN	24	0.0039789
	Transcription Factor Target	V$NRSF_01	59	0
	miRNA Target	GTATTAT,MIR-369-3P	88	0
S100A3	Kinase Target	Kinase_LYN	25	0
	Transcription Factor Target	V$SRF_01	22	0
	miRNA Target	AGGTGCA,MIR-500	42	0
S100A4	Kinase Target	Kinase_LCK	21	0
	Transcription Factor Target	V$SRF_01	22	0
	miRNA Target	ACTGCCT,MIR-34B	83	0
S100A6	Kinase Target	Kinase_LCK	23	0
	Transcription Factor Target	V$NRSF_01	55	0
	miRNA Target	TAGCTTT,MIR-9	82	0
S100A8	Kinase Target	Kinase_LYN	26	0
	Transcription Factor Target	V$NRSF_01	49	0
	miRNA Target	TGCACGA,MIR-517A,MIR-517C	7	0.097901
S100A9	Kinase Target	Kinase_LCK	21	0
	Transcription Factor Target	V$NRSF_01	58	0
	miRNA Target	GGTAACC,MIR-409-5P	10	0.0725
S100A10	Kinase Target	Kinase_LYN	22	0
	Transcription Factor Target	STTTCRNTTT_V$IRF_Q6	78	0
	miRNA Target	CAATGCA,MIR-33	35	0
S100A11	Kinase Target	Kinase_LYN	23	0
	Transcription Factor Target	STTTCRNTTT_V$IRF_Q6	68	0
	miRNA Target	ACTGCCT,MIR-34B	72	0
S100A13	Kinase Target	Kinase_CDK2	112	0
	Transcription Factor Target	V$MAX_01	76	0
	miRNA Target	GGCCAGT,MIR-193A,MIR-193B	28	0
S100A14	Kinase Target	Kinase_ATR	41	0
	Transcription Factor Target	V$E2F_01	24	0
	miRNA Target	GCAAAAA,MIR-129	63	0
S100A16	Kinase Target	Kinase_ATM	45	0.0024242
	Transcription Factor Target	GCCATNTTG_V$YY1_Q6	133	0
	miRNA Target	TAGCTTT,MIR-9	64	0

### Association between immune cell infiltration and S100A family genes in LGG

**Figure 5:**
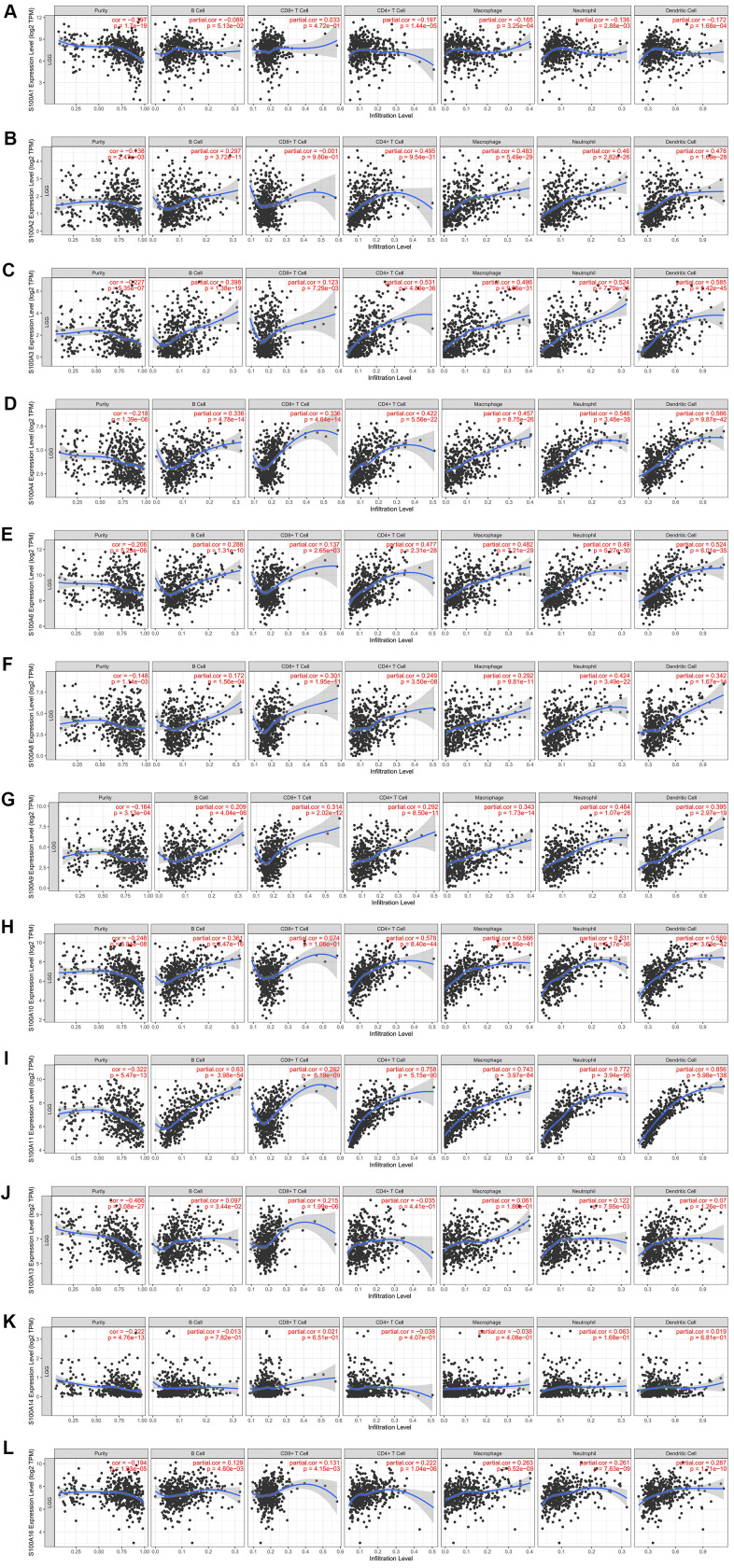
**Immune cell infiltration of S100A family genes in LGG.** (**A**) S100A1, (**B**) S100A2, (**C**) S100A3, (**D**) S100A4, (**E**) S100A6, (**F**) S100A8, (**G**) S100A9, (**H**) S100A10, (**I**) S100A11, (**J**) S100A13, (**K**) S100A14, (**L**) S100A16.

S100A family genes could influence the prognosis of LGG patients by participating in immune cell infiltration. Therefore, we investigated the correlation between S100A family genes (S100A1, S100A2, S100A3, S100A4, S100A6, S100A8, S100A9, S100A10, S100A11, S100A13, S100A14, S100A16) and immune cell infiltration via the TIMER database. S100A1 expression was negatively correlated with infiltration of CD4+ T cells (r = -0.197, p = 1.44e-05), macrophages (r = -0.165, p = 3.25e-04), neutrophils (r = -0.136, p = 2.88e-03), and dendritic cells (r = -0.172, p = 1.68e-04) (Figure 5A). S100A2 expression was positively correlated with infiltration of B cells (r = 0.297, p = 3.72e-11), CD4^+^ T cells (r = 0.495, p = 9.54e-31), macrophages (r = 0.483, p = 5.49e-29), neutrophils (r = 0.46, p = 2.82e-26), and dendritic cells (r = 0.478, p = 1.69e-28) (Figure 5B). The expression of S100A3, S100A4, S100A6, S100A8, S100A9, S100A11, and S100A16 was positively correlated with B-cell, CD8+ T-cell, CD4+ T-cell, macrophage, neutrophil, and dendritic cell infiltration (all p<0.05, Figure 5C–5G, 5I, 5L). Expression of S100A10 was positively correlated with infiltration of all immune cell types with the exception of CD8+ T cells (p < 0.05, Figure 5H). S100A13 expression was positively correlated with CD8+ T-cell infiltration (r = 0.215, p = 1.99e-06, Figure 5J). S100A14 expression was uncorrelated with immune cell infiltration (all p > 0.05, Figure 5K). We also evaluated the association between S100A family genes and immune cell infiltration with Cox proportional risk model. The results showed that LGG patient prognosis was significantly correlated with expression of S100A6 (p = 0.003), S100A10 (p = 0.001), S100A11 (p = 0.019), and S100A16 (p = 0.016, Table 3).

**Table 3:** Cox proportional risk model for the association of S100A family genes with six types of immune infiltrating cells in LGG.

	**coef**	**HR**	**95%CI_l**	**95%CI_u**	**p.value**	**sig**
B_cell	0.813	2.254	0.004	1253.711	0.801	
CD8_Tcell	5.853	348.304	0.351	346002.895	0.096	•
CD4_Tcell	1.071	2.918	0.001	12272.314	0.801	
Macrophage	4.050	57.387	0.650	5067.284	0.076	•
Neutrophil	-5.688	0.003	0.000	7.290	0.146	
Dendritic	-0.631	0.532	0.006	51.317	0.787	
S100A1	-0.083	0.920	0.776	1.091	0.339	
S100A2	-0.220	0.802	0.555	1.160	0.242	
S100A3	-0.056	0.946	0.755	1.185	0.629	
S100A4	-0.143	0.867	0.677	1.110	0.257	
S100A6	0.622	1.863	1.233	2.815	0.003	**
S100A8	0.159	1.172	0.817	1.683	0.389	
S100A9	-0.165	0.848	0.564	1.275	0.428	
S100A10	-0.405	0.667	0.524	0.850	0.001	**
S100A11	0.528	1.695	1.089	2.638	0.019	*
S100A13	0.201	1.222	0.929	1.608	0.152	
S100A14	-0.236	0.790	0.458	1.362	0.396	
S100A16	0.376	1.456	1.072	1.979	0.016	*

*P < 0.05, **P < 0.01.

### Survival analysis of S100A family genes in LGG

**Figure 6:**
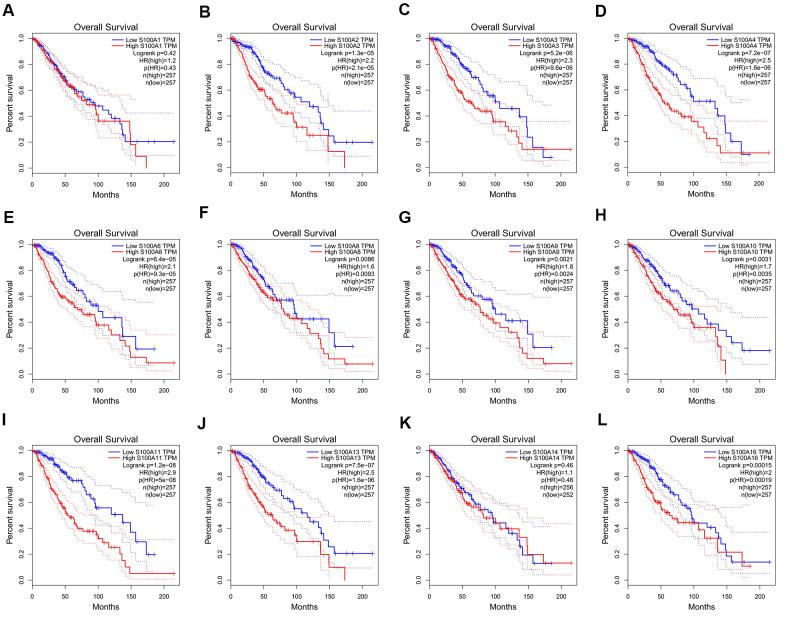
**Relationship between the gene expression level of S100A family members and overall survival of LGG patients.** (**A**) S100A1, (**B**) S100A2, (**C**) S100A3, (**D**) S100A4, (**E**) S100A6, (**F**) S100A8, (**G**) S100A9, (**H**) S100A10, (**I**) S100A11, (**J**) S100A13, (**K**) S100A14, (**L**) S100A16.

We analyzed the relationship of S100A family genes with DFS and overall survival in LGG patients based on GEPIA. Results showed that differences in the expression of S100A2, S100A3, S100A4, S100A6, S100A8, S100A9, S100A10, S100A11, S100A13, and S100A16 were significantly associated with the overall survival of LGG patients, and the high expression of forementioned genes could be a risk factor for poor prognosis of LGG patients (Figure 6A–6L).

### Correlation analysis and prognostic value analysis of S100A family in LGG

**Figure 7:**
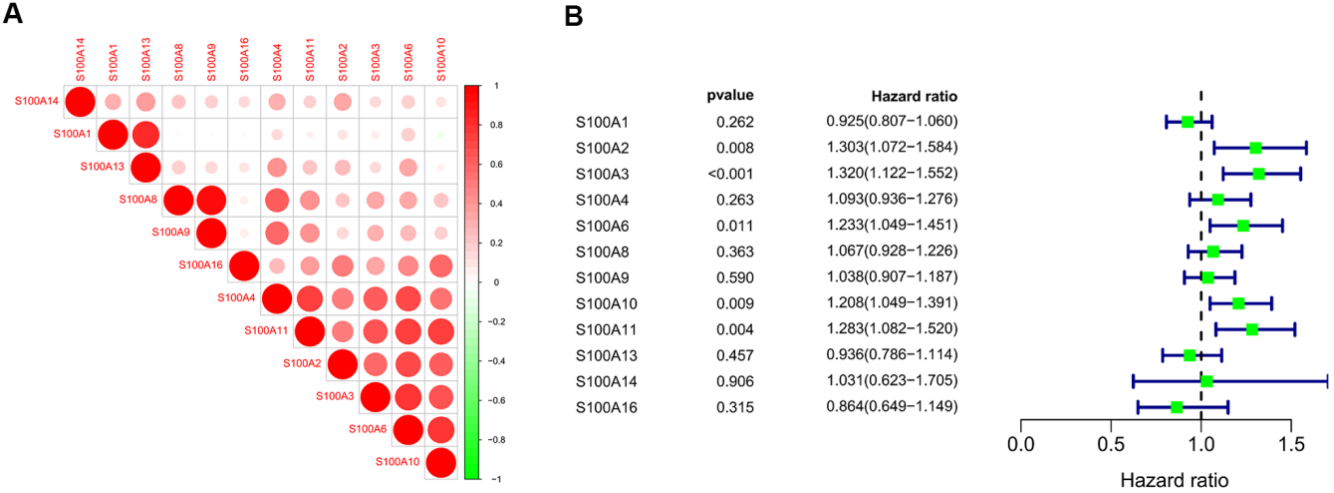
(**A**) Correlation analysis of S100A family genes of LGG samples in CGGA; (**B**) COX regression analysis of S100A family genes of LGG samples in CGGA.

We performed correlation analysis of S100A family genes in CGGA LGG samples with the R corrplot package, and found a significant positive correlation between S100A family genes (Figure 7A). S100A2, S100A3, S100A6, S100A10, and S100A11 were subsequently identified as possible prognosis-related genes in LGG patients by COX regression analysis (Figure 7B).

**Figure 8:**
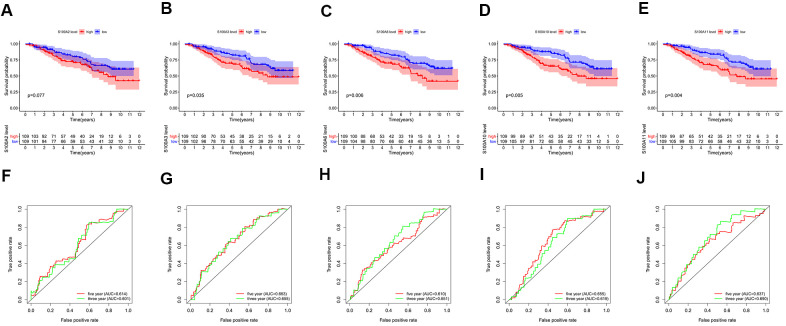
Survival analysis for high and low expression groups of prognosis-related genes: (**A**) S100A2, (**B**) S100A3, (**C**) S100A6, (**D**) S100A10, (**E**) S100A11. ROC curves for 3-year and 5-year survival of prognosis-related genes: (**F**) S100A2, (**G**) S100A3, (**H**) S100A6, (**I**) S100A10, (**J**) S100A11.

Survival analysis revealed that statistically significant differences between the high and low expression groups of S100A3, S100A6, S100A10, and S100A11 genes, and high expression groups of S100A3, S100A6, S100A10, and S100A11 genes may be a risk factor of poor prognosis for LGG patients (P<0.05, Figure 8A–8E).

**Figure 9:**
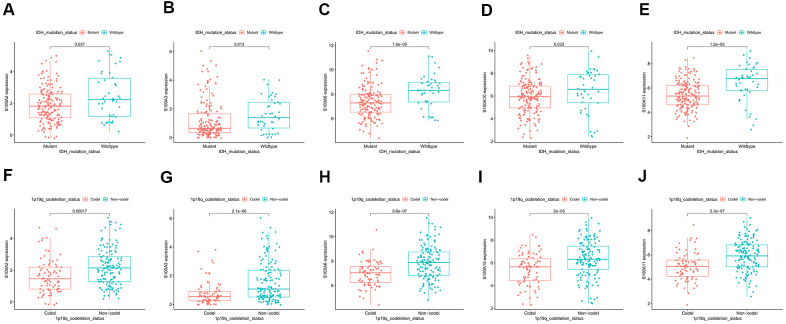
Correlation of prognosis-related gene expression in IDH Mutant and Wildtype LGG: (**A**) S100A2, (**B**) S100A3, (**C**) S100A6, (**D**) S100A10, (**E**) S100A11. Correlation of prognosis-related gene expression in 1p19q Codeletion and Non-Codeletion LGG: (**F**) S100A2, (**G**) S100A3, (**H**) S100A6, (**I**) S100A10, (**J**) S100A11.

The AUC (area under curve) values of the 3-year and 5-year survival ROC curves for S100A2, S100A3, S100A6, S100A10, and S100A11 were >0.6, indicating moderate accuracy for predicting LGG prognosis (Figure 8F–8J). Clinical correlation analysis showed that the expression of S100A2, S100A3, S100A6, S100A10, and S100A11 was significantly lower in IDH Mutant and 1p19q codeletion LGG than in wildtype and 1p19q non-codeletion (Figure 9A–9J).

### Immunohistochemistry

**Figure 10:**
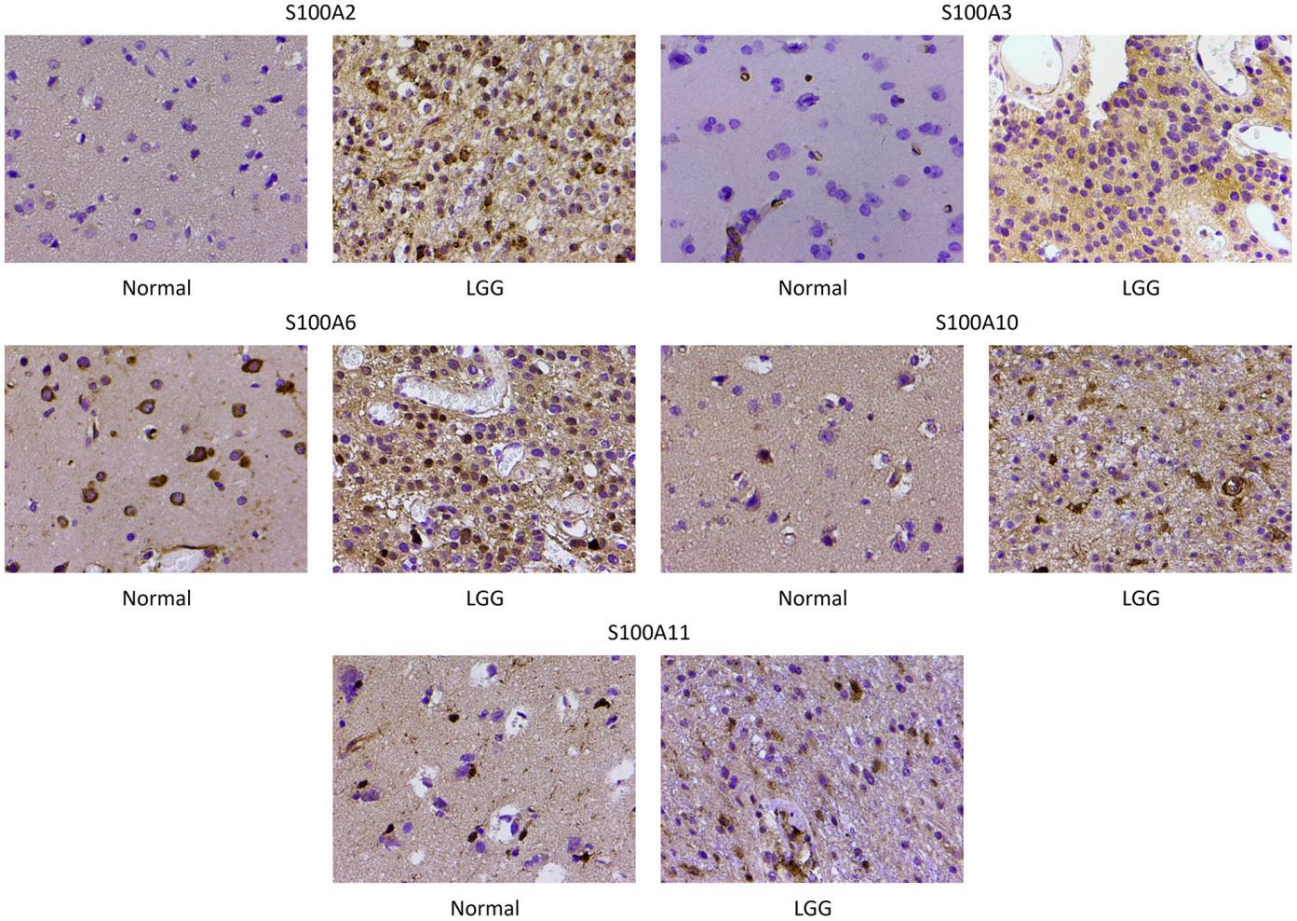
**Immunohistochemical staining of S100A2, S100A3, S100A6, S100A10 and S100A11 in normal brain and low-grade glioma.** Magnification, ×200.

The immunohistochemical staining validated the previous database analyses indicating that S100A2, S100A3, S100A6, S100A10, and S100A11 expression was upregulated in LGGs. The results revealed that S100A2, S100A3, S100A6, S100A10, and S100A11 were primarily expressed in the cytoplasm of cells, and S100A2, S100A3, S100A6, S100A10, and S100A11 expression was significantly higher in LGG compared with normal brain tissue (Figure 10).

## DISCUSSION

Although scientists have recognized numerous well-known immune regulatory sites, such as cell death protein 1 (PD-1), cell death ligand 1 (PDL-1), and cytotoxic T lymphocyte antigen 4 (CTLA4), monogenetic therapies are still ineffective [28, 29]. Thus, in the current study, we explored additional driver genes exerting immunosuppression. Calcium-binding activity of S100A family members was noted during our analysis. For instance, S100A4 has been reported to promote malignant progression of glioma [27].

The S100A protein family consists of 16 members, which regulate cell proliferation and differentiation, Ca2+ homeostasis, and inflammation among other processes [30]. During the early stages of inflammation, interleukin-1α (IL-1α), interleukin-33 (IL-33), and S100A family proteins function together in the regulation and warning of inflammation [31, 32]. Once released into the extracellular space, specific S100A proteins modulate innate and acquired immune responses, direct cell migration and chemotaxis, and induce tissue development and repair by interacting with various receptors [20, 30, 31].

The S100A family genes are differentially expressed in many tumors and have been detected in multiple tumors over the years. For example, through a comprehensive analysis of information from multiple patients, S100A2 was identified as a potential predictor of breast cancer [33]. Breast cancer studies showed that S100A2 is a tumor suppressor gene that is primarily regulated by BRCA1/p63 and plays a role in regulating the stability of mutant p53 [34]. In squamous cell carcinoma, FADU and RPMI-2650 cell lines showed high and low levels of S100A2, respectively, and S100A2 expression had a significant inhibitory effect on cell activity [35]. Glucose transporter type 1 (GLUT1) plays an important role in the process of glycolysis. Studies showed that activation of the S100A2/GLUT1 axis can promote colon cancer progression by regulating the glycolytic process [36].

In the analysis of Wang et al., S100A3 was found to be a potential predictive marker for gastric cancer [37]. Some researchers reported that inhibiting the expression of S100A3 significantly reduced the invasion ability of prostate cancer and inhibited tumor growth [38]. In hepatocellular carcinoma (HCC), S100A3 expression is associated with tumorigenesis and tumor aggressiveness [39]. The pharmacological activity of all trans-retinoic acid (ATRA) is in part mediated by retinoic acid receptor (RAR) transcription factors. S100A3 knockdown reduced the amount of RARα in breast and lung cancer cells, and thus induced resistance to ATRA differentiation, suggesting that S100A3 is an important regulatory factor affecting breast and lung cancer cell differentiation [40]. However, the role of S100A2 and S100A3 in brain tumors has been less well studied.

S100A family proteins are important regulators of immune-related biological behavior in glioma cell lines. Macrophages contribute to immune defense, immunomodulation, and tissue repair processes [41]. Depending on the conditions of cytokine production and activation, macrophages can be divided into two categories: pro-inflammatory M1 (classically activated macrophages) and anti-inflammatory M2 (alternatively activated macrophages) [42, 43]. By facilitating angiogenesis and immune evasion, M2-type macrophages can promote glioma progression [44]. In tumor biology, macrophages tend to differentiate into the M2 type rather than the tumor-killing M1 phenotype and produce cytokines such as IL-10, IL-4, and IL-13, which can promote tumor progression [45, 46]. Several members of the S100A family have macrophage regulatory functions. In periodontitis, S100A12 expression is higher in classical monocytes than in non-classical monocytes. In the process of differentiation from monocytes to macrophages, the expression and protein secretion of S100A12 is significantly decreased [47]. In a mouse model of peritonitis, S100A10 is directly involved in inflammation-stimulated plasminogen dependent macrophage recruitment [48]. Dulyaninova Ng et al. established a S100A4-deficient mouse model, and they found that S100A4 can regulate macrophage invasion through myosin-dependent and independent mechanisms [49]. Moreover, S100A4 can regulate the recruitment and chemotaxis of macrophages in mice [50].

S100A4 is an upstream regulator of epithelial-mesenchymal transition (EMT) master regulators SNAIL2 and ZEB, as well as other mesenchymal transition regulators of glioblastoma. Tumors with high S100A4 expression present higher tumor initiation and spheroid ability [27]. In glioma, S100A4 expression is affected by DNA methylation, β-linked proteins, and extracellular factors including epidermal growth factor and tumor necrosis factor alpha (TNF-α) [51]. By binding to calcium, S100A4 affects tumor cell motility and metastasis. Moreover, as the grade of glioma increases, S100A4 expression also increases [52]. S100A4 protein serves as a direct signaling target of receptor tyrosine kinase 2 (ERBB2) in medulloblastoma through a pathway involving phosphatidylinositol 3 kinase AKT (PI3K/AKT), which is ultimately blocked by the ERBB tyrosine kinase inhibitor OSI774 [53].

The infiltrating state of neutrophils is closely related to glioma development [54, 55]. Centrogranulocytes can promote the progression and growth of glioma through S100A4 [56]. S100A4 protein alters the expression of transcription factors and signal transduction pathway genes involved in T cell differentiation. Studies found that in S100A4-transfected T cells, the proportion of Th1-polarized cells was reduced and the Th1/Th2 balance shifted towards a Th2 tumorigenic phenotype [57]. Also, the expression of S100A4 and S100A6 was significantly increased, indicating a strong correlation between them [58].

S100A6 has a tumor-promotional effect in a variety of cancers. In colorectal and cervical cancer, S100A6 stimulates the proliferation and migration of cancer cells through the mitogen-activated protein kinases (MAPK) and PI3K/AKT signaling pathways, respectively [59, 60]. Moreover, S100A6 can regulate acetylation of P53 gene, thereby regulating the activity of lung cancer cells [61]. In an earlier study, changes in S100A6 protein expression levels were markers of differentiation between low-grade astrocytic tumors [62]. In conclusion, S100A6 is differentially expressed in a variety of cancers, and detection of serum S100A6 levels may aid in cancer diagnosis [18].

S100A8 and S100A9 proteins are potential immune modulators. They are involved in the regulation of cyclin expression [63]. S100A8 regulates activation of monocyte toll-like receptor 4 (TLR4) and controls the development of immune processes [64, 65]. Bone marrow-derived immunosuppressive cells were found to greatly hinder immune recognition of glioma cells [66]. High expression of S100A8 and S100A9 is strongly linked to tumor promotion [67–69]. High expression of S100A8 and S100A9 in glioma inhibits T cell function and their differentiation via interferon-alpha (INF-α) to regulate production of macrophages or dendritic cells [70, 71]. Furthermore, S100A8 and S100A9 are constitutively expressed in neutrophils and monocytes as Ca(2+) sensors and are involved in cytoskeletal rearrangement and arachidonic acid metabolism. By stimulating leukocyte recruitment and inducing cytokine secretion, they regulate inflammatory responses [72]. Acute-phase response protein serum amyloid SAA1 and SAA3 regulate the transcription of S100A4 through the TLR4/NF-κB signaling pathway. They can moderate transcription of S100A8 and S100A9 proteins and exert immunomodulatory functions [73].

The cancer-promoting effect of S100A10 is obvious in ovarian cancer [74, 75], the protein also stimulates production of breast cancer stem cells [76]. S100A10 mediates macrophage migration to tumor sites and increases fibrosarcoma invasion [77]. However, investigations of the role of S100A10 in brain tumor immunity are scarce.

S100A11 is known to be associated with poor prognosis in glioma patients. S100A11 upregulation can activate the NF-kB pathway and stimulate the invasion and migration of glioma. S100A11, whose associations with Annexin A2 (AXNA2) have been demonstrated, is suggested to be involved in regulation of the cell cycle [78].

In conclusion, multiple S100A family protein members contribute to tumor progression via their close relationship with inflammation.

## CONCLUSIONS

Through the verification of multiple databases, we observed important roles of the inflammatory S100A family in glioma. Immunoinfiltration analysis showed that expression of multiple genes in the S100A family was highly correlated with the infiltration state of macrophages, neutrophils, and dendritic cells. There is considerable evidence of the correlation between S100A4 and glioma. There are relatively few studies on S100A2 and S100A3 genes in glioma immunity, and there is no sufficient evidence to show their specific role in glioma immunity at present. S100A6 has a tumor-promoting effect in a variety of cancers, and S100A6 expression may be an important factor to distinguish glioma grade, potentially in serum. In addition, S100A8, S100A9, S100A10, and S100A11 genes also showed a strong correlation with immune infiltration. In our data, S100A10 and S100A11 had the strongest correlation with the immune cell infiltration in glioma. Therefore, we believe that the immunomodulatory effect of the S100A family members is an important factor affecting the progression of glioma.

## MATERIALS AND METHODS

**Figure 11:**
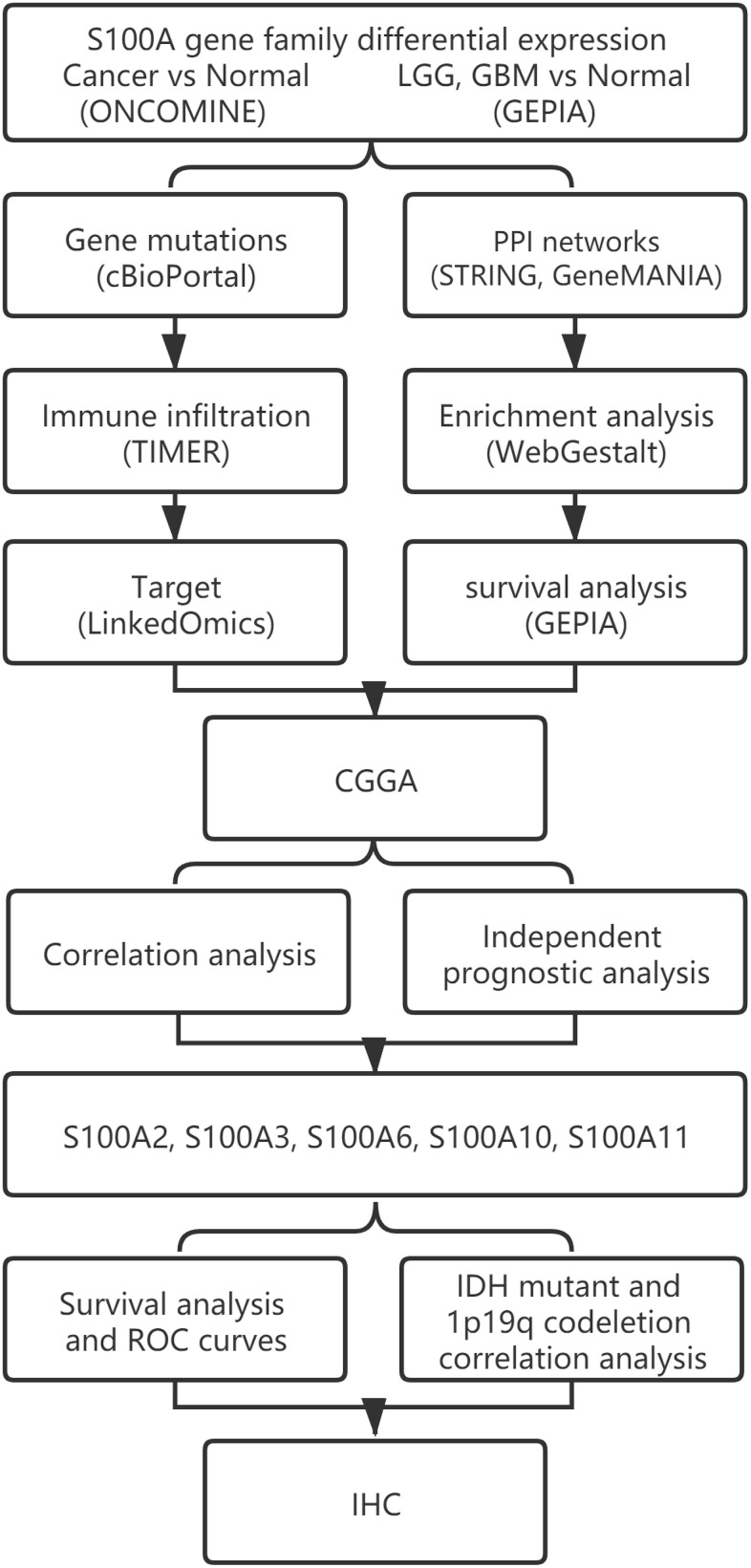
The workflow diagram of this study.

A study flowchart is presented in Figure 11.

### ONCOMINE

Oncomine (https://www.oncomine.org) is a large-scale oncogene microarray database, covering 65 gene microarray datasets, 4,700 microarrays, and expression data for 480 million genes, which can be used for analyzing gene expression differences, finding outliers, predicting co-expressed genes, etc. Data from Oncomine can be classified based on clinical information such as tumor stage, grade, and tissue type. We retrieved the mRNA expression data of S100A family genes in tumor tissue and normal tissue from database. In our analysis, p < 0.05, 2-fold change, and a top 10% gene rank were set as thresholds.

### GEPIA

The GEPIA database (http://gepia.cancer-pku.cn/index.html) integrated TCGA cancer profiles and GTEx normal profiles to address important questions in cancer biology. With bioinformatics methods, we can reveal cancer subtypes, driver genes, alleles, and differentially expressed or carcinogenic factors, enabling in-depth exploration of novel cancer targets and markers. We employed the GEPIA Single Gene Analysis module and analyzed mRNA expression of the S100A gene family in LGG, GBM, and normal tissues. Finally, we used the survival analysis module to assess relations between S100A family gene expression and overall survival in LGG patients to plot survival curves. Hazard ratios were calculated based on the Cox PH (proportional hazard) model, the 95% confidence interval (CI) is indicated by dashed lines, the x-axis unit is months, and *p* < 0.05 is considered statistically significant.

### cBioPortal

cBioPortal (https://www.cbioportal.org) is a comprehensive open web platform based on the TCGA database which integrated data mining, data integration, and visualization. We obtained mutation profile and genetic variation of the S100A family in 511 LGG samples (Samples with log2 copy-number data) from Brain Lower Grade Glioma (TCGA, PanCancer Atlas). mRNA expression was z-scored relative to all samples (log RNA Seq V2 RSEM), and a z-score threshold was set at ±2.

### String

The String database (https://string-db.org/) is a searchable tool of known and predictive protein interactions in 2031 species, which includes 9.6 million proteins and 13.8 million protein interactions. It contains experimental data, results from text mining of PubMed abstracts, data synthesized from other databases, and predicted results via bioinformatics methods. We pictured protein–protein interaction (PPI) networks of the S100A gene family via the String database.

### GeneMANIA

GeneMANIA (http://www.genemania.org) was used to predict protein interactions and to analyze co-expression, co-localization, pathways, physical interactions of the S100A gene family. Website prediction and other profiles were used to explore the potential protein functions of genes.

### WebGestalt

WebGestalt (http://www.webgestalt.org/) is a widely used set of gene set enrichment analysis tools for functional enrichment analysis in different biological contexts. It is a powerful integrated data mining system capable of managing, retrieving, organizing, visualizing, and statistically analyzing large amounts of genes. We performed Gene ontology (GO) functional and KEGG pathway enrichment analysis of S100A family genes and protein network-related genes of GeneMANIA via WebGestalt's Over-Representation Analysis (ORA). GO annotations are divided into three major categories: Biological Process (BP), Cellular Components (CC), and Molecular Function (MF). The KEGG pathway was designed to identify the various pathways involved in the function of individual genes. The screening criteria were set at significance level of TOP10. The FDR value was set at 0.05.

### LinkedOmics

The LinkedOmics database (http://www.linkedomics.org/) consists of multi-omics and clinical profiles from 32 cancer types, as well as 11,158 patient profiles from the Cancer Genome Atlas (TCGA) project. We studied kinase target, transcription factor target and miRNA target of the S100A gene family based on the Gene Set Enrichment Analysis (GSEA) of the LinkInterpreter module. Gene Set Enrichment Analysis (GSEA) was conducted under the following criteria: Rank Criteria (from LinkFinder Result) is the FDR value, Minimum Number of Genes (Size) is 3, and Simulations is 500.

### TIMER

TIMER (https://cistrome.shinyapps.io/timer/) is a web tool created by Harvard University. It provided the profile of six types of infiltrating immune cells (B cells, CD4+ T cells, CD8+ T cells, neutrophils, macrophages, and dendritic cells) in tumor tissues which was detected by RNA-Seq expression profiling data. We assessed the correlation of S100A family expression levels with immune cell infiltration, LGG patient survival via the TIMER gene module, and survival module based on the Cox proportional risk model.

### CGGA and R (4.0.2)

The CGGA database (http://www.cgga.org.cn/) is a database including brain tumor datasets from a Chinese cohort of more than 2000 samples. It contains whole exome sequencing, DNA methylation, mRNA sequencing, mRNA and microRNA microarrays, and matched clinical data. R is an open software programming language and operating environment for statistical analysis, mapping, and data mining. In this study, based on data from 218 LGGs with mRNAseq 693 and mRNAseq 325 samples in CGGA, we performed correlation analysis of S100A family genes using R 4.0.2. We also screened LGG prognosis-related genes via COX regression models, and their accuracy as prognostic genes was verified by survival analysis of high and low expression groups, Receiver Operating Characteristic (ROC) Curve analysis, and clinical correlation analysis.

### Immunohistochemistry

Immunohistochemical staining was used to detect the expression of prognosis-related genes in normal brain and LGG tissues. The experiments utilizing human tissue were approved by the ethics committee of the First Hospital of Shanxi Medical University. Five samples of normal brain tissue in patients with epilepsy and traumatic brain injury, and five LGG samples were collected from the First Hospital of Shanxi Medical University. All postoperative tissues were examined pathologically in the Department of Pathology, First Hospital of Shanxi Medical University. After routine paraffin-embedding, tissue sections were obtained, placed on glass microscope slides, de-paraffinized, and rehydrated. Antigen retrieval and blocking of endogenous peroxidases were performed, followed by exposure to corresponding gene polyclonal antibodies (Sangon, Shanghai, China) and enzyme-labeled IgG polymers. Finally, antibodies were visualized using a diaminobenzidine (DAB) chromogenic solution and hematoxylin as a counterstain.

### Ethics approval and consent to participate

This human tissue study was reviewed and approved by the Ethics Committee of the First Hospital of Shanxi Medical University (K042-2020-04-03), and patients provided written informed consent to participate in this study.

### Availability of data and material

All data are available from ONCOMINE, GEPIA, cBioPortal, String, GeneMANIA, WebGestalt, LinkedOmics, TIMER and CGGA databases.
